# Determination of the optimal target level of proteinuria in the management of patients with glomerular diseases by using different definitions of proteinuria

**DOI:** 10.1097/MD.0000000000008154

**Published:** 2017-11-03

**Authors:** Youn Kyung Kee, Chan-Yun Yoon, Seung Jun Kim, Sung Jin Moon, Chan Ho Kim, Jung Tak Park, Beom Jin Lim, Tae Ik Chang, Ea Wha Kang, Jeong Hae Kie, Tae-Hyun Yoo, Hyun Joo Jeong, Shin-Wook Kang, Seung Hyeok Han

**Affiliations:** a Department of Internal Medicine, College of Medicine, Institute of Kidney Disease Research, Yonsei University; b Department of Internal Medicine, International St. Mary's Hospital, Catholic Kwandong University; c Department of Pathology, College of Medicine, Institute of Kidney Disease Research, Yonsei University; d Department of Internal Medicine, NHIS Medical Center, Ilsan Hospital, Seoul, South Korea.

**Keywords:** focal segmental glomerulosclerosis, IgA nephropathy, membranous nephropathy, outcome, proteinuria

## Abstract

Proteinuria is a major determinant of adverse renal outcome, and its reduction slows renal progression in glomerular diseases. However, the optimal target of proteinuria in glomerular diseases is unclear, and discrepancies in the definition of proteinuria produce ambiguous findings. Here we investigated the optimal target of proteinuria by using different definitions of proteinuria. We analyzed 574 IgA nephropathy (IgAN), 175 membranous nephropathy (MGN), and 177 focal segmental glomerulosclerosis (FSGS) cases from 3 Korean kidney centers. We evaluated the impact of proteinuria on renal outcome with 2 definitions: time-average proteinuria (TAP) and time-varying proteinuria (TVP). The endpoint was renal progression, defined as a 50% decline in glomerular filtration rate or end-stage renal disease. During a median follow-up of 57.3 months, the primary outcome occurred in 54 patients with IgAN, 26 with MGN, and 30 with FSGS. Multivariate Cox regression using TAP indicated that there was a linear association between proteinuria and risk of renal progression in IgAN. However, moderate proteinuria was not associated with an increased risk of renal progression in MGN and FSGS. In contrast, the analysis by TVP showed that the risk significantly increased in proportion to proteinuria during follow-up in all 3 diseases. Our findings suggest that TVP-based model can delineate association between proteinuria and risk of renal progression better than TAP-based model, considering that TVP reflects the dynamic change of proteinuria over time. Thus, proteinuria reduction to the lowest possible level is required to improve renal outcomes in patients with glomerular diseases.

## Abstract 2

Supplemental Digital Content is available in the text

## 1 Introduction

Glomerular disease is an important cause of end-stage renal disease (ESRD)^[1]^ and has a variable clinical course, from a benign status without any renal impairment to rapid progression to ESRD.^[2–7]^ Proteinuria is the hallmark of glomerular damage, and high urinary protein excretion is known to be related to an increased risk of renal progression.^[8,9]^ In addition, the level of proteinuria has been used to monitor responses to treatment, as the reduction of proteinuria is associated with a decreased risk of progression of kidney diseases.^[10]^ In this regard, it is important to reduce the amount of proteinuria to the lowest possible level in order to prevent the adverse outcomes of glomerular disease.^[11]^ For example, we and other groups have consistently shown that proteinuria reduction to a level of <1.0 g/day or 1.0 g/g creatinine results in the lowest risk of progression of immunoglobulin A nephropathy (IgAN).^[12–14]^ However, a complete disappearance of proteinuria is difficult to achieve, and residual proteinuria inevitably persists in many patients despite aggressive immunosuppressive treatment. In fact, there is no clearly defined target level of proteinuria reduction in glomerular diseases. Interestingly, the progression of kidney disease seems to differ even at the same level of proteinuria depending on the type of primary glomerular disease. Cattran et al^[15]^ evaluated renal progression in patients with membranous glomerulonephropathy (MGN), focal segmental glomerulosclerosis (FSGS), and IgAN, and found discrepant rates of renal function decline in those with similar proteinuria levels. Notably, different definitions of proteinuria have been used in previous studies of glomerular diseases.^[15–18]^ These include baseline proteinuria, time-average proteinuria (TAP), and time-varying proteinuria (TVP). For variables that can change over time, measurements of fixed variables at a certain time point may be inappropriate for assessing their risk during the whole disease course. In fact, a recent study suggested that TVP can reflect the dynamic alteration of proteinuria over time, and thus is the optimal parameter for determining the prognostic effects of proteinuria.^[19]^

Therefore, the purpose of this study was to investigate the optimal target for proteinuria in the management of various glomerular diseases, including IgAN, MGN, and FSGS by using 2 different definitions of proteinuria: TAP and TVP. In addition, we evaluated which definition of proteinuria is more informative for the risk stratification of these glomerular diseases.

## 2 Materials and methods

### 2.1 Patient selection

**Figure 1:**
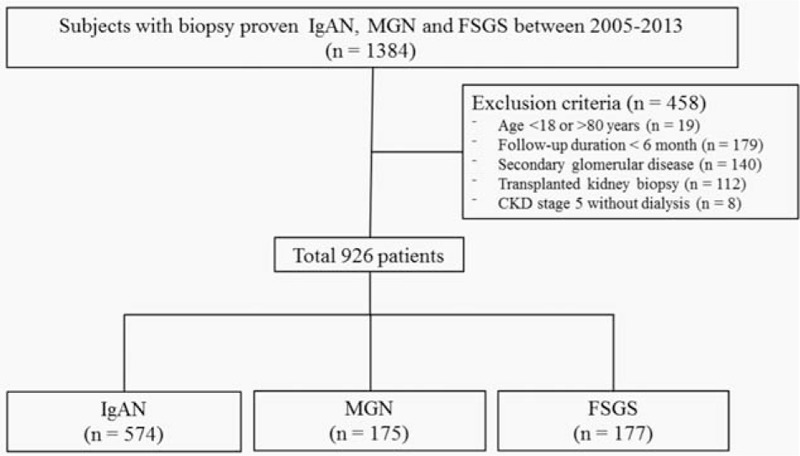
Study population. FSGS = focal segmental glomerulosclerosis, IgAN = immunoglobulin A nephropathy, MGN = membranous glomerulonephritis.

A flowchart depicting the selection of subjects is presented in Fig. 1. A total of 1416 renal biopsies had been performed in 1384 patients between 2005 and 2013 in 3 Korean kidney centers: Yonsei University Severance Hospital (n = 1003, 70.8%), International St. Mary's Hospital (n = 113, 8.0%), and National Health Insurance Corporation Ilsan Hospital (n = 300, 21.2%). All of our subjects were Koreans, and we excluded 490 patients who met the following criteria: age <18 or >80 years at presentation (n = 19), follow-up duration <6 months (n = 179), secondary causes of glomerular disease including diabetes (n = 172), transplanted kidney biopsies (n = 112), or chronic kidney disease (CKD) stage 5 without dialysis (n = 8). Renal biopsies were performed by medical judgment of each nephrologist from 3 kidney centers. Second biopsy was performed in 32 patients, when either a considerable worsening of clinical course or a change in treatment strategy was considered. In these patients, data from the first biopsy were included in the analysis. Patients with missing data were also excluded. Therefore, a total of 934 patients with primary glomerular diseases were finally included in the analysis (IgAN, n = 574; MGN, n = 175; FSGS, n = 177). The study was approved by the Institutional Review Board (IRB) of Yonsei University Health System Clinical Trial Center. Because the present study is a retrospective observational study and the study subjects were anonymized, the IRB waived the need for written consent from the patients.

### 2.2 Data collection

Baseline demographic data including sex, age, systolic blood pressure (SBP), diastolic blood pressure (DBP), body mass index, history of hypertension, and laboratory data were collected at the time of renal biopsy. Mean arterial pressure (MAP) was determined as the DBP plus one-third of the pulse pressure. The collected laboratory data were as follows: white blood cell count, hemoglobin, glucose, albumin, calcium, phosphate, blood urea nitrogen, creatinine, total cholesterol, and estimated glomerular filtration rate (eGFR). Medications such as renin–angiotensin system (RAS) blockers, corticosteroids, and other immunosuppressants (cyclosporine and cyclophosphamide) were also investigated. Users of RAS blockers were defined as patients who received these medications for ≥3 months. Immunosuppressive treatment with corticosteroid or other immunosuppressants was considered to treat regardless of the duration of therapy. Proteinuria during follow-up was assessed by using the spot urine protein–creatinine ratio (UPCr), and the eGFR of each patient was calculated using the Chronic Kidney Disease Epidemiology Collaboration (CKD-EPI) equation.^[20]^ The UPCr and eGFR values were determined every 3 months.

### 2.3 Definition

Definition of various proteinuria metrics is well described elsewhere.^[19]^ In short, TAP is defined as the average of proteinuria values measured at each 3-month interval from renal biopsy to the end of follow-up or occurrence of primary outcome. Follow-up time is divided into time window of 3-month and TVP is the value measured each time window. Detailed description on how to handle time-varying covariates is beyond the scope of this study and has been published elsewhere.^[21,22]^

### 2.4 Treatment

As a retrospective design, the management of patients could not be standardized and fully controlled, but the institutions treated the patients with glomerular diseases according to general guideline-based protocol. Intensive conservative therapy, such as administration of RAS blockers and diet restriction, was preferentially given for BP control and reduction of proteinuria in all 3 types of glomerular diseases. For IgAN patients who had persistent proteinuria >1.0 g/g creatinine, corticosteroids were administered. We generally followed the 6-month corticosteroid treatment protocol as previously suggested by Pozzi et al.^[23]^ For MGN patients, the risk of progression was assessed according to the Kidney Disease Improving Global Outcomes guideline.^[24]^ Immunosuppression was postponed up to 6 months after the diagnosis in patients with a low or moderate risk whose serum creatinine was maintained stably and urinary protein excretion did not persistently exceed 4 g/d declining over 50% of baseline value during an observation period with antihypertensive and antiproteinuric therapy. For patients with a high risk or those with a moderate risk and rapid progression, corticosteroids plus cyclosporine or cyclophosphamide were initiated. The typical protocol for high-risk MGN was the Ponticelli regimen, as previously suggested.^[25]^ Patients with FSGS who had features of nephrotic syndrome were initially treated with oral prednisolone at a single dose of 1 mg kg^−1^ day^−1^, with gradual tapering of dosage if CR or PR was achieved. Cyclosporine or cyclophosphamide was added in cases of frequent relapses, incomplete responses, or intolerance to corticosteroids.

### 2.5 Study endpoint

The patients were followed up until December 31, 2015. The time “0” of this study was the time of renal biopsy before initiating treatment. The study primary endpoint was a sustained decrease in eGFR of >50% for at least 2 consecutive measurements, or the onset of ESRD. ESRD was defined as the initiation of chronic dialysis, or kidney transplantation. Renal survival times were defined as the duration from the time of biopsy to the last follow-up.

### 2.6 Statistical analysis

Normally distributed variables are expressed as mean ± standard deviation and compared by using 1-way analysis of variance. Categorical variables were compared by the Chi-square test, as required. Cox proportional hazard models were used to examine the associations of TAP and TVP with renal progression. All analyses were stratified by glomerulonephritis type. The association of TAP grouped by 4 categories with renal outcome was estimated. In time-varying models, proteinuria and BP were calculated and updated at each quarter during the entire follow-up, to assess the short-term associations between proteinuria and renal outcome.^[19,21,26]^ Clinically relevant factors or significantly associated variables with renal outcomes in univariate analyses were adjusted in multivariate models. The results are expressed as a hazard ratio and 95% confidential interval. Death that occurred before reaching primary outcome was treated as a competing risk. The discriminatory ability of each proteinuria metric was compared by C-statistics. The rate of renal function decline per year was assessed by using the slope of eGFR obtained from a generalized linear mixed model. Data were analyzed by using SPSS version 23 software (SPSS, Chicago, IL) and SAS version 9.2 (SAS, Inc., Cary, NC). All *P*-values were 2-tailed, and a value of <.05 was considered statistically significant.

## 3 Results

### 3.1 Patient characteristics according to types of glomerular disease

The patient characteristics according to types of glomerular disease are shown in Table 1. Patients with MGN (55.0 ± 15.1 years) were older than those with IgAN (38.5 ± 13.1 years) or FSGS (47.3 ± 17.0 years). There was no sex difference among the groups. Blood pressure (BP) was significantly higher and the presence of hypertension was more common in patients with FSGS than in the other 2 groups. In contrast, immunosuppressant therapy was more commonly used in patients with MGN and FSGS than in those with IgAN. The mean follow-up duration was 61.3, 50.7, and 54.0 months for IgAN, MGN, and FSGS, respectively. The baseline UPCr was significantly higher in patients with MGN (5.37 ± 4.17 g/g) and FSGS (4.26 ± 5.45 g/g) than in those with IgAN (1.42 ± 1.84 g/g), whereas the baseline eGFR was significantly higher in patients with IgAN (88.2 ± 32.9 mL min^−1^ 1.73 m^−2^) and MGN (87.7 ± 36.9 mL min^−1^ 1.73 m^−2^) than in those with FSGS (79.7 ± 40.8 mL min^−1^ 1.73 m^−2^).

**Table 1:**
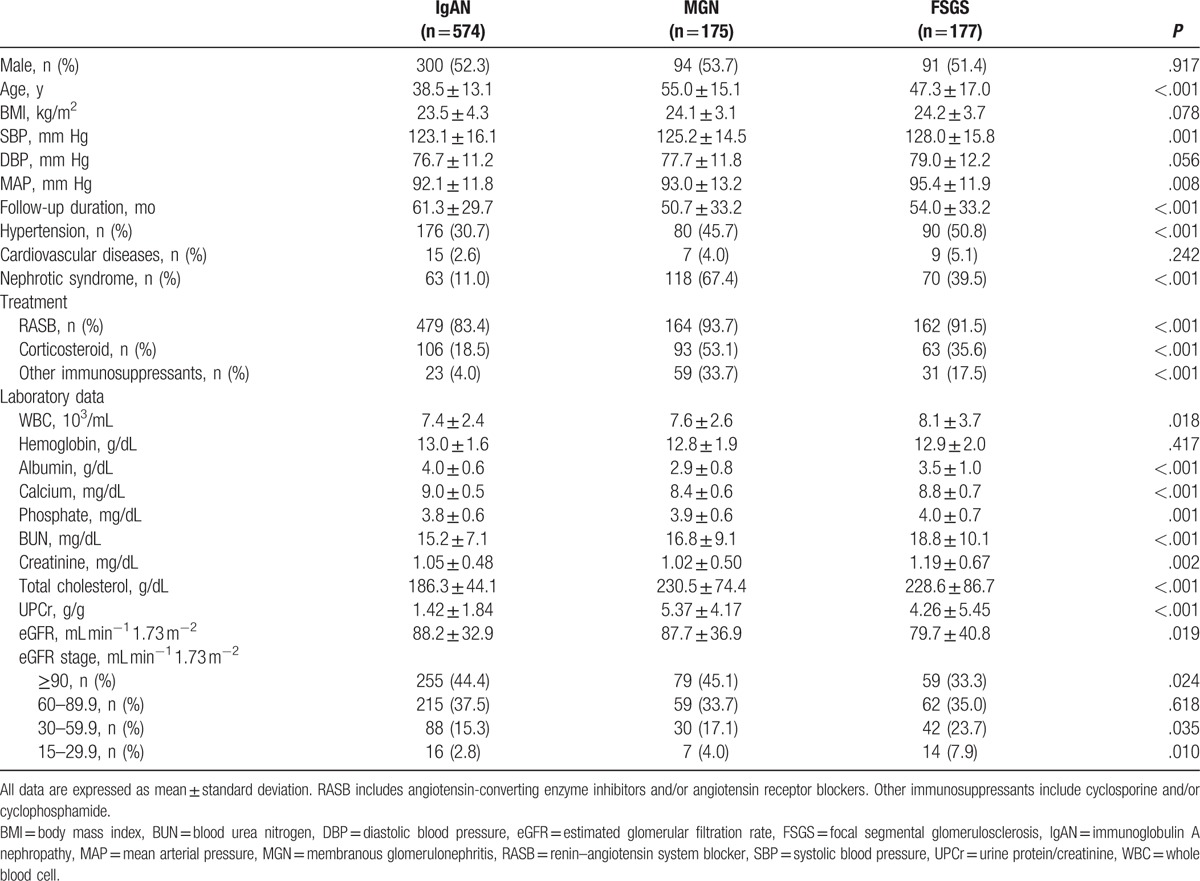
Baseline characteristics of the study patients according to the types of glomerular disease.

We analyzed 574 patients with IgAN, 175 with MGN, and 177 with FSGS. All patients were categorized into 4 groups according to TAP (<1.0, 1.0–1.99, 2.0–2.99, and ≥3.0 g/g). In patients with IgAN, SBP, and MAP showed an increasing tendency as TAP increased (*P* for trend = .049 and .043), whereas there was no difference in these variables between the TAP groups in patients with MGN (*P* for trend = .437 and .054) and FSGS (*P* for trend = .321 and .331). Patients with higher TAP tended to have lower levels of eGFR at the time of biopsy and serum albumin in IgAN (all *P* for trend <.001) and FSGS (*P* for trend = .043 and <.001). In addition, these patients were more commonly treated with immunosuppressive agents than those with lower TAP (*P* < .001 for IgAN and FSGS, *P* = .011 for MGN). The detailed descriptions of baseline characteristics according to the 4 categories of TAP in each group of glomerular disease are presented in Supplemental Table S1.

### 3.2 Renal outcomes according to 4 categories of TAP

**Figure 2:**
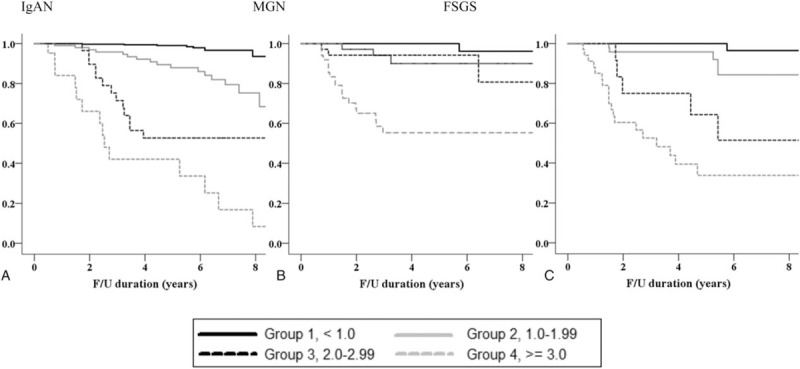
Kaplan–Meier renal survival curves based on 4 categories of time-average proteinuria. FSGS = focal segmental glomerulosclerosis, IgAN = immunoglobulin A nephropathy, MGN = membranous glomerulonephritis.

Table 2 shows the renal outcomes of the study patients according to TAP in each type of glomerular disease. Among 926 patients, primary outcome occurred in 110 (11.9%) patients (54 [9.4%] with IgAN, 26 [14.9%] with MGN, and 30 [16.9%] with FSGS) during a median follow-up duration of 57.3 (6.2–127.6) months. No patients progressed to ESRD before reaching a 50% decline in eGFR. There were graded increases in the development of the primary outcome as TAP increased in all types of glomerular disease. For example, in the IgAN group, 9 (2.2%), 17 (16.0%), 13 (43.3%), and 15 (71.4%) patients with TAP of <1.0, 1.0 to 1.99, 2.0 to 2.99, and ≥3.0 g/g reached a 50% decline in eGFR or ESRD (*P* = .001). Similar patterns were observed in patients with MGN and FSGS. Kaplan–Meier curves in IgAN patients also showed that the renal-event-free survival was significantly decreased in groups with a higher TAP. However, in MGN patients with TAP of 1.0 to 1.99 and 2.0 to 2.99 g/g, renal survival was similar to those with TAP of <1.0 g/g, whereas it was markedly decreased in those with TAP of ≥3.0 g/g. Similarly, the renal survival rate did not differ between FSGS patients with TAP of 1.0 to 1.99 g/g and those with TAP of <1.0 g/g (Fig. 2).

**Table 2:**
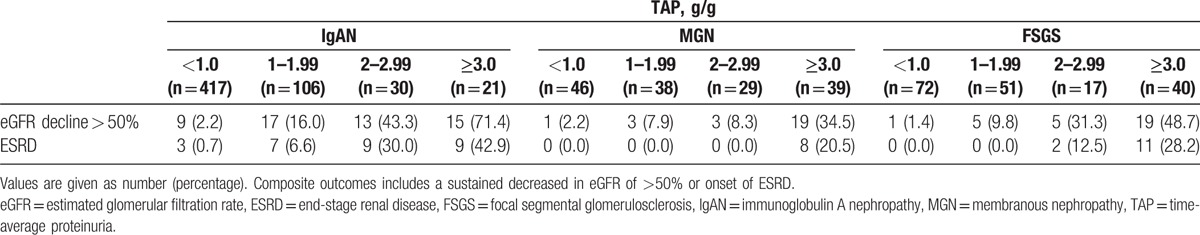
Renal outcomes according to 4 categories of time-average proteinuria in 3 types of glomerular disease.

### 3.3 Optimal target level of proteinuria assessed according to TAP and TVP

**Figure 3:**
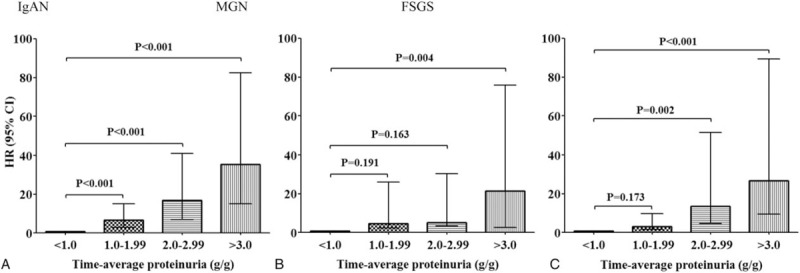
Risks for renal progression based on 4 categories of time-average proteinuria (reference group: TAP < 1.0 g/g). All variables were adjusted for age, sex, mean arterial pressure, steroid and immunosuppressant use, and baseline estimated glomerular filtration rate. CI = confidential interval, FSGS = focal segmental glomerulosclerosis, HR = hazard ratio, IgAN = immunoglobulin A nephropathy, MGN = membranous glomerulonephritis, TAP = time-average proteinuria.

To determine the association between the risk for renal progression and the level of proteinuria, 2 different definitions of proteinuria were used in the Cox proportional hazard models. Table 3 presents both unadjusted and adjusted Cox models with TAP and TVP. We adjusted factors that showed either significant association with renal outcome in univariate analyses or well-established factors related to renal outcome. In time-varying TVP models, time-varying MAPs calculated at each quarter over the entire follow-up period were used for adjustment. Patients with TAP or TVP < 1.0 g/g were set as reference group in all Cox analyses. In the analyses with TAP, the risk of renal progression significantly increased in groups with higher TAP. In particular, IgAN patients with TAP <1.0 g/g had the lowest risk of renal progression, as compared with those with TAP ≥ 1.0 g/g. The HRs for reaching the primary outcome were 6.72 (95% CI: 2.96–15.25, *P* < .001), 16.39 (95% CI: 6.69–40.16, *P* < .001), and 30.84 (95% CI: 13.04–72.95, *P* < .001) in patients with TAP of 1.0 to 1.99, 2.0 to 2.99, and ≥3.0 g/g, respectively. Similar associations were observed in patients with MGN and FSGS. However, in these patients with moderate proteinuria levels of 1.0 to 2.99 g/g, the risk of renal progression was not significantly greater than in those with TAP < 1.0 g/g. In MGN patients with TAP of 1.0 to 1.99 (HR 4.63, 95% CI: 2.47–25.99, *P* = .191) and 2.0 to 2.99 g/g (HR 5.09, 95% CI: 3.52–30.28, *P* = .163), and in FSGS patients with TAP of 1.0 to 1.99 g/g (HR 6.43, 95% CI: 1.12–13.12, *P* = .093), the risk of renal progression did not differ from those with TAP < 1.0 g/g (Fig. 3). We confirmed these associations by comparing the rates of renal function decline among the 4 TAP groups. In patients with IgAN, those with TAP of 1.0 to 1.99 g/g (−0.59 ± 0.24 mL min^−1^ 1.73 m^−2^) had a faster decline in eGFR than those with TAP < 1.0 g/g (0.42 ± 0.24 mL min^−1^ 1.73 m^−2^, *P* < .001). However, in MGN and FSGS patients, there was no difference in the rates of eGFR decline between those with TAP of 1.0 to 1.99 g/g and those with TAP < 1.0 g/g (Supplementary Table S2).

**Table 3:**
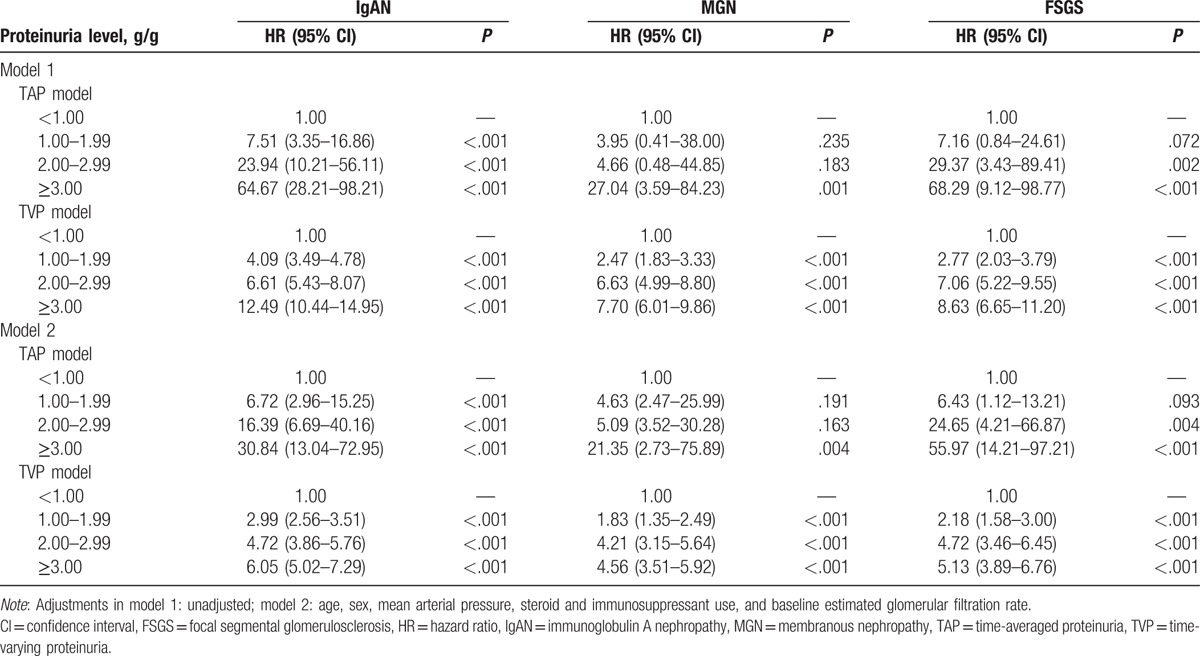
Association of TAP and TVP with kidney disease progression.

**Figure 4:**
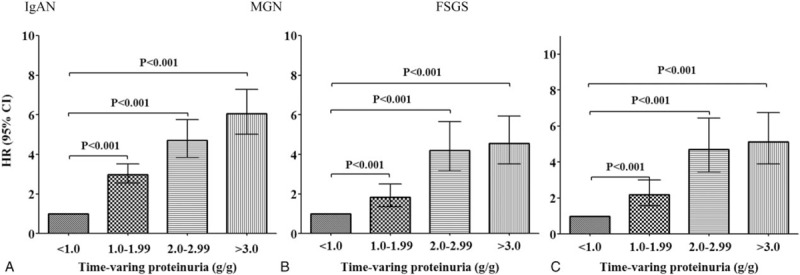
Risks for renal progression based on 4 categories of time-varying proteinuria (reference group: TVP < 1.0 g/g). All variables were adjusted for age, sex, mean arterial pressure, steroid and immunosuppressant use, and baseline estimated glomerular filtration rate. CI = confidential interval, FSGS = focal segmental glomerulosclerosis, HR = hazard ratio, IgAN = immunoglobulin A nephropathy, MGN = membranous glomerulonephritis, TVP = time-varying proteinuria.

**Figure 5:**
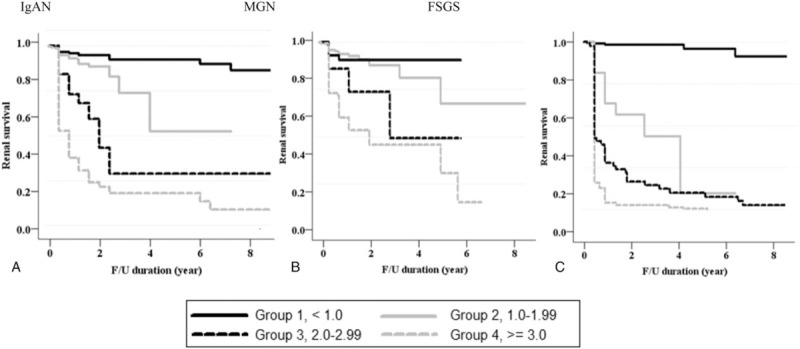
Kaplan–Meier renal survival curves based on 4 categories of time-varying proteinuria. FSGS = focal segmental glomerulosclerosis, IgAN = immunoglobulin A nephropathy, MGN = membranous glomerulonephritis.

Next, we applied time-varying Cox models in which proteinuria and BP were treated as time-varying covariates (Fig. 4). As in the TAP-based analyses, in patients with IgAN, there were significantly graded increases in the risk of renal progression in higher TVP groups. The HRs for developing the primary outcome in patients with IgAN were 2.99 (95% CI: 2.56–3.51, *P* < .001), 4.72 (95% CI: 3.86–5.76, *P* < .001), and 6.05 (95% CI: 5.02–7.29, *P* < .001) for patients with TVP of 1.0 to 1.99, 2.0 to 2.99, and 3.0 g/g, respectively. In contrast to the analyses with TAP, the risk of renal progression in MGN and FSGS patients with moderate proteinuria levels of 1.0 to 2.99 g/g was significantly increased as compared with those with TVP of <1.0 g/g. In particular, MGN patients with TVP of 1.0 to 1.99 g/g had a 1.8-fold increased risk of reaching the primary outcome (95% CI: 1.40–2.57, *P* < .001). In addition, FSGS patients with TVP of 1.0 to 1.99 g/g were also significantly associated with an increased risk of developing the primary outcome (HR 2.18, 95% CI: 1.58–3.00, *P* < .001). Kaplan–Meier curves also confirmed these associations. The renal survival rates were significantly decreased in patients with TVP of 1.0 to 1.99 g/g compared with those with TVP < 1.0 g/g in all 3 groups of glomerular disease (Fig. 5). In additional analyses with TAP and TVP normalized to body surface area, there was no difference in the overall results and there was no improvement in predictive ability (Supplementary Table S3).

### 3.4 Additive prognostic value of proteinuria metrics for renal outcome prediction

To confirm the validity of TVP and TAP as a useful biomarker, we compared C-statistics between TVP-based and TAP-based multivariate Cox regression models. All ΔC-statistics of TVP-based models were significantly higher than those of TAP-based models (Table 4). This finding suggested that TVP predicted renal outcome better than TAP.

**Table 4:**
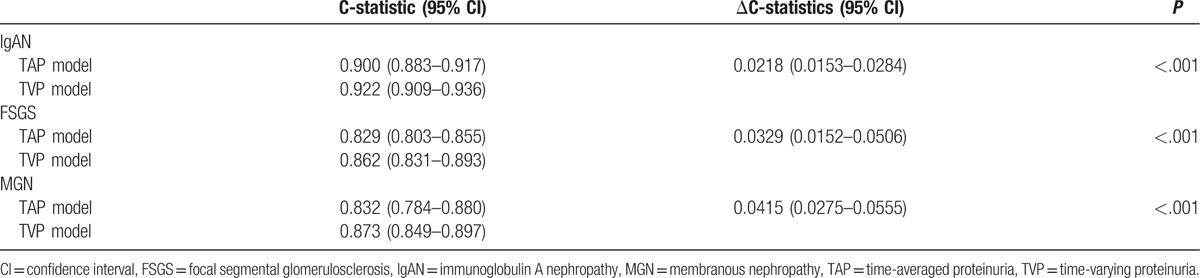
Comparing the prognostic value for prediction of renal outcomes according to TAP and TVP.

## 4 Discussion

In this study, we evaluated the optimal target level of proteinuria in patients with the 3 most common types of glomerular disease using 2 different definitions of proteinuria. In IgAN, both TAP- and TVP-based analyses indicated that patients with proteinuria <1.0 g/g were at the lowest risk of renal progression than those with proteinuria ≥1.0 g/g. However, in the MGN and FSGS groups, patients with moderate proteinuria levels of 1.0 to 2.99 g/g were not associated with the development of adverse renal outcome when analyzed using TAP. Interestingly, by using time-varying Cox models, we clearly showed that the risk of renal progression was minimized when proteinuria was decreased to <1.0 g/g in patients with MGN and FSGS. Thus, by using TVP, this study substantiated the importance of proteinuria reduction in the management of various glomerular diseases, supporting the concept of “the lower, the better”.

Glomerular disease is the third most common cause of ESRD worldwide. IgAN, MGN, and FSGS are the most frequently diagnosed types of glomerular disease, and can progress to ESRD unless complete remission (CR) is achieved.^[27]^ Similar to other kidney diseases, baseline eGFR, pathologic features, response to therapy, and persistent proteinuria are strongly associated with renal progression in patients with glomerular disease.^[28–30]^ In particular, sustained proteinuria correlates well with adverse renal outcomes in these patients, and the degree of proteinuria has been used to monitor therapeutic response and to predict patient outcome.^[8,31,32]^ Therefore, proteinuria reduction should undoubtedly be incorporated into the treatment strategies to prevent the renal progression. However, complete disappearance of proteinuria does not occur in all patients and residual proteinuria persists in many patients despite the proper use of immunosuppressive agents. In fact, CR is achieved in only 30% to 70% of patients with MGN^[33,34]^ and in 20% to 30% of patients with FSGS.^[35–37]^ To date, the relationship between the risks of residual proteinuria and future outcome has not yet been clarified.

In this regard, previous findings indicating the importance of achieving partial remission (PR) in MGN and FSGS should be considered. The serial studies demonstrated that patients who attained at least PR had better renal survival than those who had no remission; however, patients with CR had the best survival.^[17,38]^ Nevertheless, these studies did not investigate the optimal level of proteinuria for preventing renal progression. Only 1 study thus far has evaluated this issue. In a study by Cattran et al,^[15]^ proteinuria of 1.0 to 2.99 g 1.73 m^−2^ day^−1^ was not associated with deterioration of kidney function compared with proteinuria of 0.5 to 1.0 g 1.73 m^−2^ day^−1^ in MGN and FSGS, whereas the risk of progression started to increase from proteinuria of >1.0 g 1.73 m^−2^ day^−1^ in IgAN. In particular, there was no difference in the rate of renal function decline between MGN patients with proteinuria 3.0 to 5.0 g 1.73 m^−2^ day^−1^ and those with proteinuria of 0.5 to 1.0 g 1.73 m^−2^ day^−1^. According to this finding, the target level of proteinuria seems to differ depending on the type of glomerular disease. However, the finding should be interpreted with caution because TAP was used in the study. In fact, baseline proteinuria and TAP have been criticized because these parameters cannot capture longitudinal changes in proteinuria over time. To overcome this limitation of fixed covariates, many epidemiologic studies have recently utilized a time-varying Cox model. By calculating the weighted average of all the time-window-specific HRs or risk ratios, this model enables carrying out more precise risk assessments particularly when the covariates are highly variable during follow-up. Barbour et al recognized the strength of TVP and analyzed the renal outcome in 1351 adults with IgAN, MGN, and FSGS, by using various definitions of proteinuria. They found that TVP best accounted for the prognostic effects of proteinuria over time, whereas biased results were produced by other metrics up to 30.3%.^[19]^ Considering these strengths and shortcomings of the different definitions of proteinuria, we sought to estimate optimal target level of proteinuria to delay renal progression in 3 glomerular diseases by applying both TAP and TVP. In the analysis with TAP, patients with IgAN had an increased risk for renal progression in proportion to the increasing level of proteinuria during treatment, even at a proteinuria level of 1.0 to 2.0 g/g, whereas renal progression was not observed in patients with MGN or FSGS having proteinuria level up to 3.0 g/g. In contrast, TVP-based analyses clearly showed that even moderately increased proteinuria of 1.0 to 2.99 g/g was significantly associated with a markedly increased risk of renal progression, suggesting that the target proteinuria level should be lowered to <1.0 g/g in all 3 glomerular diseases. Because TVP can overcome the shortcoming of baseline proteinuria and TAP which are unable to reflect the high variability of proteinuria during follow-up, we believe that TVP-based analysis is the ideal method to assess the risk of being persistently exposed to proteinuria, as suggested by Barbour et al.^[19]^ It may be difficult to apply this method to clinical practice immediately, as it requires multiple measurements during longitudinal follow-up period. However, by doing this, TVP metric can be helpful in determining an optimal level of proteinuria target to achieve the best clinical outcomes. Nevertheless, well-designed randomized controlled trials are recommended to solve such complex issue.

This study has several limitations. First, we could not analyze whether further reduction of proteinuria to <0.5 g/g would be more helpful for improving renal outcomes. A Chinese cohort study by Le et al showed that IgAN patients with TAP of 0.5 to 1.0 g/day had an increased risk of renal progression compared with those with TAP of <0.5 g/day. In line with this finding, our previous study also showed a faster decline rate of eGFR in IgAN patients with TAP of 0.3 to 1.0 g/g than in those with TAP of <0.3 g/g.^[13]^ In addition, several studies have shown that CR conferred better renal outcomes than PR.^[17,39]^ However, patients with proteinuria <1.0 g/day are unlikely to develop renal events compared with those with proteinuria >1.0 g/day, which can result in a lack of statistical power. Further long-term studies in large cohorts are required to explore the prognostic implications of minimal proteinuria. Second, pathologic findings were not included in the analyses because different pathologic classification systems are used for the 3 glomerular diseases. Some pathologic features such as tubulointerstitial fibrosis are strongly associated with deterioration of kidney function.^[40–43]^ However, tubulointerstitial lesion is incorporated only in the Oxford classification of IgAN. The Columbia classification of FSGS includes 5 morphologic subtypes, and the pathologic classification of MGN mainly describes electron dense deposits in the glomerular basement membrane. Nevertheless, we separately analyzed IgAN patients by adding the Oxford classification in the time-varying Cox model, and found that adjustment for pathologic features did not alter the study results (data not shown). Third, the 3 glomerular diseases in this study exhibit slow progression; thus, long-term outcome studies are not clinically feasible in most research cohorts.^[44]^ Our study is also limited by the relatively small sample size, the small number of renal events, and the relatively short follow-up duration. However, as a glomerular disease cohort, the sample size and follow-up duration were not inferior to those in the TORONTO registry,^[45]^ which is the largest cohort of glomerulonephritis worldwide. Further studies with larger sample sizes and longer follow-up duration are required to consolidate our results and to clarify the long-term effect of residual proteinuria on future outcomes in patients with glomerular disease. Fourth, there was no preset indication for renal biopsy in our cohort. In most cases, it was determined by physicians based on the presented signs, symptoms, and laboratory findings of patients. However, it is well known that the prevalence, biopsy practice pattern, treatment, and renal survival rate vary depending on regions.^[46]^ In addition, in some glomerulonephritis ethnic difference can impact on disease progression.^[47]^ This geographic variability and ethnic difference could not be reflected in our observational study.

In conclusion, caution should be exercised when determining the optimal target level of proteinuria and the risk of renal progression because the results can differ depending on which definition of proteinuria is applied in the analysis. Given the strengths of TVP, we believe that this is an appropriate method to identify the effect of continuously changing proteinuria on renal outcomes over time. By using this method, this study showed that the risk for renal progression was increased in proportion to the level of proteinuria during the follow-up period in 3 different glomerular diseases. Therefore, proteinuria reduction to the lowest possible level may be helpful to improve renal outcomes in patients with glomerular diseases.

## Supplementary Material

Supplemental Digital Content
