# Supplementary material for: Determination of the optimal target level of proteinuria in the management of patients with glomerular diseases by using different definitions of proteinuria
Source: Medicine (Baltimore). 2017 Nov 3;96(44):e8154. doi: 10.1097/MD.0000000000008154 (PMC5682769; doi:10.1097/MD.0000000000008154)
Supplement: Supplemental Digital Content [file medi-96-e8154-s001.docx]

<Supplementary Materials>

***Table S1-A.* Baseline characteristics of patients with immunoglobulin A nephropathy according to four categories of time-average proteinuria**

|  | TAP (g/g) | | | | *P* value |
| --- | --- | --- | --- | --- | --- |
|  | <1.0 | 1.0-1.99 | 2.0-2.99 | >3.0 |  |
|  | (n=417) | (n=106) | (n=30) | (n=21) |  |
| Male (n, %) | 219 (52.5) | 56 (52.8) | 14 (46.7) | 11 (52.4) | 0.940 |
| Age (years) | 37.7 ± 13.1 | 38.3 ± 11.2 | 46.2 ± 13.9 | 42.6 ± 17.5 | <0.001 |
| BMI (kg/m^2^) | 23.5 ± 4.6 | 23.6 ± 3.3 | 23.4 ± 3.1 | 24.8 ± 3.2 | 0.621 |
| SBP (mmHg) | 122.8 ± 15.3 | 121.9 ± 15.3 | 129.5 ± 20.5 | 128.2 ± 23.7 | 0.049 |
| DBP (mmHg) | 76.3 ± 11.4 | 76.2 ± 10.5 | 80.9 ± 12.5 | 80.2 ± 7.8 | 0.071 |
| MAP (mmHg) | 91.8 ± 11.8 | 91.4 ± 11.3 | 97.1 ± 14.3 | 96.6 ± 10.2 | 0.043 |
| Follow-up duration (month) | 62.7 ± 28.8 | 62.8 ± 30.5 | 54.3 ± 31.0 | 35.6 ± 30.3 | <0.001 |
| Hypertension (n, %) | 108 (25.9) | 40 (37.7) | 15 (50.0) | 13 (61.9) | <0.001 |
| *Treatment* |  |  |  |  |  |
| RASB (n, %) | 328 (78.7) | 102 (96.2) | 28 (93.3) | 21 (100.0) | <0.001 |
| Corticosteroid (n, %) | 52 (12.5) | 30 (28.3) | 12 (40.0) | 12 (57.1) | <0.001 |
| Other immunosuppressants (n, %) | 10 (2.4) | 6 (5.7) | 6 (20.0) | 1 (4.8) | <0.001 |
| *Laboratory data* |  |  |  |  |  |
| WBC (10^3^/mL) | 7.3 ± 2.2 | 7.7 ± 2.3 | 7.9 ± 2.0 | 8.3 ± 4.9 | 0.058 |
| Hemoglobin (g/dL) | 13.1 ± 1.6 | 13.0 ± 1.7 | 12.3 ± 1.8 | 11.6 ± 1.6 | <0.001 |
| Albumin (g/dL) | 4.1 ± 0.5 | 4.0 ± 0.5 | 3.6 ± 0.7 | 3.3 ± 0.6 | <0.001 |
| Calcium (mg/dL) | 9.1 ± 0.5 | 9.0 ± 0.5 | 8.9 ± 0.5 | 8.6 ± 0.5 | <0.001 |
| Phosphate (mg/dL) | 3.8 ± 0.6 | 3.8 ± 0.5 | 3.8 ± 0.7 | 4.1 ± 0.6 | 0.057 |
| BUN (mg/dL) | 14.3 ± 5.8 | 15.8 ± 6.4 | 20.5 ± 13.5 | 21.9 ± 13.7 | <0.001 |
| Creatinine (mg/dL) | 0.99 ± 0.39 | 1.11 ± 0.46 | 1.39 ± 0.79 | 1.48 ± 0.94 | <0.001 |
| Total cholesterol (g/dL) | 182.2 ± 44.4 | 193.5 ± 42.5 | 207.8 ± 40.3 | 200.66 ± 35.4 | 0.001 |
| UPCr (g/g) | 0.9 ± 1.1 | 2.1 ± 2.1 | 3.3 ± 3.0 | 5.2 ± 3.0 | <0.001 |
| eGFR (mL⋅min^-1^⋅1.73 m^-2^) | 92.6 ± 32.4 | 81.1 ± 30.1 | 70.1 ± 34.6 | 61.3 ± 32.9 | <0.001 |

<Supplementary Materials>

***Table S1-B.* Baseline characteristics of patients with membranous glomerulonephritis according to four categories of time-average proteinuria**

|  | TAP (g/g) | | | | *P* value |
| --- | --- | --- | --- | --- | --- |
|  | <1.0 | 1.0-1.99 | 2.0-2.99 | >3.0 |  |
|  | (n = 46) | (n = 38) | (n = 36) | (n = 55) |  |
| Male (n, %) | 24 (52.2) | 17 (44.7) | 21 (58.3) | 32 (58.2) | 0.567 |
| Age (years) | 52.0 ± 14.4 | 51.2 ± 14.1 | 53.7 ± 15.3 | 61.0 ± 14.8 | 0.004 |
| BMI (kg/m^2^) | 24.6 ± 2.7 | 24.0 ± 3.7 | 23.6 ± 3.2 | 24.0 ± 2.9 | 0.553 |
| SBP (mmHg) | 123.8 ±16.9 | 128.2 ± 12.2 | 124.0 ± 15.7 | 125.2 ± 12.9 | 0.527 |
| DBP (mmHg) | 75.9 ± 11.9 | 78.6 ± 9.9 | 79.3 ± 15.8 | 77.6 ± 9.9 | 0.579 |
| MAP (mmHg) | 89.9 ± 18.2 | 95.1 ± 9.5 | 94.2 ± 12.9 | 93.4 ±10.1 | 0.268 |
| Follow-up duration (month) | 69.6 ± 33.6 | 55.6 ± 30.9 | 48.6 ± 30.0 | 32.8 ± 26.9 | <0.001 |
| Hypertension (n, %) | 18 (39.1) | 11 (28.9) | 20 (55.6) | 31 (56.4) | 0.029 |
| *Treatment* |  |  |  |  |  |
| RASB (n, %) | 42 (91.3) | 36 (94.7) | 34 (94.4) | 52 (94.5) | 0.892 |
| Corticosteroid (n, %) | 14 (30.4) | 24 (63.2) | 23 (63.9) | 32 (58.2) | 0.004 |
| Other immunosuppressants (n, %) | 5 (10.9) | 17 (44.7) | 16 (44.4) | 20 (36.4) | 0.002 |
| *Laboratory data* |  |  |  |  |  |
| WBC (10^3^/mL) | 7.4 ± 2.6 | 7.9 ± 3.1 | 7.5 ± 2.4 | 7.8 ± 2.5 | 0.752 |
| Hemoglobin (g/dL) | 13.2 ± 1.6 | 12.5 ± 1.9 | 12.9 ± 1.7 | 12.6 ± 2.2 | 0.240 |
| Albumin (g/dL) | 3.6 ± 0.7 | 2.9 ± 0.8 | 2.6 ± 0.7 | 2.7 ± 0.7 | <0.001 |
| Calcium (mg/dL) | 8.8 ± 0.5 | 8.3 ± 0.7 | 8.3 ± 0.5 | 8.2 ± 0.6 | <0.001 |
| Phosphate (mg/dL) | 3.9 ± 0.6 | 3.9 ± 0.7 | 3.7 ± 0.7 | 3.9 ± 0.6 | 0.429 |
| BUN (mg/dL) | 13.9 ± 5.9 | 16.0 ± 6.3 | 16.8 ± 10.1 | 19.9 ± 11.2 | 0.008 |
| Creatinine (mg/dL) | 0.9 ± 0.3 | 1.0 ± 0.5 | 1.0 ± 0.4 | 1.2 ± 0.6 | 0.033 |
| Total cholesterol (g/dL) | 206.6 ± 54.3 | 232.8 ± 76.7 | 239.5 ± 70.9 | 243.0 ± 85.8 | 0.075 |
| UPCr (g/g) | 2.1 ± 1.9 | 3.9 ± 2.0 | 7.2 ± 4.5 | 7.9 ± 4.2 | <0.001 |
| eGFR (mL⋅min^-1^⋅1.73 m^-2^) | 89.1 ± 22.1 | 97.9 ± 51.1 | 93.8 ± 36.5 | 75.4 ± 32.7 | 0.017 |

<Supplementary Materials>

***Table S1-C.* Baseline characteristics of patients with focal segmental glomerulosclerosis according to four categories of time-average proteinuria**

|  | TAP (g/g) | | | | *P* value |
| --- | --- | --- | --- | --- | --- |
|  | <1.0 | 1.0-1.99 | 2.0-2.99 | >3.0 |  |
|  | (n = 71) | (n = 51) | (n = 16) | (n = 39) |  |
| Male (n, %) | 40 (56.3) | 22 (43.1) | 8 (50.0) | 21 (53.8) | 0.533 |
| Age (years) | 44.5 ± 16.2 | 50.6 ± 16.4 | 50.7 ± 18.6 | 46.5 ± 18.2 | 0.211 |
| BMI (kg/m^2^) | 23.9 ± 2.9 | 24.5 ± 3.7 | 23.4 ± 3.3 | 24.5 ± 4.9 | 0.651 |
| SBP (mmHg) | 126.7 ± 16.4 | 126.7 ± 15.1 | 130.7 ± 11.3 | 131.6 ± 15.6 | 0.321 |
| DBP (mmHg) | 78.3 ± 12.3 | 77.8 ± 11.7 | 80.1 ± 9.4 | 81.4 ± 13.7 | 0.499 |
| MAP (mmHg) | 94.3 ± 12.2 | 94.1 ± 11.6 | 96.9 ± 8.4 | 98.1 ± 12.9 | 0.331 |
| Follow-up duration (month) | 63.5 ± 30.6 | 59.2 ± 31.1 | 48.9 ± 38.7 | 32.2 ± 28.5 | <0.001 |
| Hypertension (n, %) | 29 (40.8) | 31 (60.8) | 8 (50.0) | 22 (56.4) | 0.148 |
| *Treatment* |  |  |  |  |  |
| RASB (n, %) | 63 (88.7) | 48 (94.1) | 16 (100.0) | 35 (89.7) | 0.424 |
| Corticosteroid (n, %) | 8 (11.3) | 20 (39.2) | 11 (68.8) | 24 (61.5) | <0.001 |
| Other immunosuppressants (n, %) | 5 (7.0) | 10 (19.6) | 4 (25.0) | 12(30.8) | 0.012 |
| *Laboratory data* |  |  |  |  |  |
| WBC (10^3^/mL) | 7.6 ± 2.6 | 8.4 ± 4.4 | 9.6 ± 4.2 | 7.9 ± 3.9 | 0.212 |
| Hemoglobin (g/dL) | 13.5 ± 1.7 | 12.9 ± 2.0 | 12.5 ± 1.8 | 12.2 ± 2.0 | 0.003 |
| Albumin (g/dL) | 3.9 ± 0.8 | 3.4 ± 0.9 | 2.9 ± 0.9 | 3.0 ± 0.9 | <0.001 |
| Calcium (mg/dL) | 9.1 ± 0.6 | 8.7 ± 0.7 | 8.5 ± 0.8 | 8.4 ± 0.7 | <0.001 |
| Phosphate (mg/dL) | 4.0 ± 0.8 | 3.9 ± 0.6 | 3.8 ± 0.6 | 4.1 ± 0.7 | 0.588 |
| BUN (mg/dL) | 14.7 ± 5.2 | 19.4 ± 9.2 | 21.7 ± 12.6 | 24.6 ± 13.6 | <0.001 |
| Creatinine (mg/dL) | 0.99 ± 0.38 | 1.17 ± 0.67 | 1.18 ± 0.49 | 1.61 ± 0.94 | <0.001 |
| Total cholesterol (g/dL) | 199.5 ± 41.3 | 231.9 ± 97.3 | 251.3 ± 83.4 | 268.0 ± 114.4 | <0.001 |
| UPCr (g/g) | 1.1 ± 1.6 | 4.6 ± 4.6 | 6.6 ± 5.5 | 8.2 ± 7.4 | <0.001 |
| eGFR (mL⋅min^-1^⋅1.73 m^-2^) | 89.6 ± 29.6 | 74.0 ± 28.9 | 76.7 ± 42.6 | 70.5 ± 62.8 | 0.043 |

<Supplementary Materials>

***Table S2***. **Rates of renal function decline based on four categories of time-average proteinuria (TAP) (reference group: TAP <1.0 g/g)**

| TAP Group (g/g) | IgAN | |  | MGN | |  | FSGS | |
| --- | --- | --- | --- | --- | --- | --- | --- | --- |
|  | Slope of eGFR decline (mL/min/1.73m^2^/year) | *P* value |  | Slope of eGFR decline (mL/min/1.73m^2^/year) | *P* value |  | Slope of eGFR decline (mL/min/1.73m^2^/year) | *P* value |
| <1.0 | 0.42 ± 0.08 (0.26 to 0.58) | ref. |  | 0.48 ± 0.28 (0.09 to 0.96) | ref. |  | 0.56 ± 0.31 (-0.45 to 1.16) | ref. |
| 1.0-1.99 | -0.59 ± 0.24 (-1.06 to -0.11) | <0.001 |  | 0.45 ± 0.42 (-0.38 to 1.29) | 0.427 |  | 0.41 ± 0.24 (-0.52 to 0.89) | 0.722 |
| 2.0-2.99 | -1.53 ± 0.13 (-1.79 to -1.28) | <0.001 |  | -0.20 ± 0.27 (-0.90 to 0.49) | <0.001 |  | -1.55 ± 0.44 (-2.41 to -0.68) | <0.001 |
| ≥3.0 | -4.15 ± 0.45 (-5.06 to -3.24) | <0.001 |  | -2.71 ± 0.38 (-3.46 to -1.96) | <0.001 |  | -3.20 ± 0.39 (-5.45 to -0.40) | <0.001 |

**Table S3. Comparing the prognostic value for prediction of renal outcomes according to BSA-normalized TAP and TVP**

|  |  | C-statistic (95% CI) | Δ C-statistics (95% CI) | *P* value |
| --- | --- | --- | --- | --- |
| IgAN | TAP model | 0.887 (0.872 – 0.901) | 0.013 (0.004 – 0.021) | <0.001 |
|  | TVP model | 0.900 (0.887 – 0.917) |  |  |
| FSGS | TAP model | 0.834 (0.803 – 0.865) | 0.035 (0.027 – 0.044) | <0.001 |
|  | TVP model | 0.869 (0.840 – 0.897) |  |  |
| MGN | TAP model | 0.805 (0.776 – 0.835) | 0.038 (0.030 – 0.046) |  |
|  | TVP model | 0.843 (0.818 – 0.869) |  | <0.001 |
| *Abbreviations:* TAP, time-averaged proteinuria; TVP, time-varying proteinuria; IgAN, IgA nephropathy; MGN, membranous nephropathy; FSGS, focal segmental glomerulosclerosis; CI, confidence interval | | | | |
